# Neural systems supporting cognitive-affective interactions in adolescence: the role of puberty and implications for affective disorders

**DOI:** 10.3389/fnint.2012.00065

**Published:** 2012-08-31

**Authors:** Cecile D. Ladouceur

**Affiliations:** Department of Psychiatry, University of PittsburghPittsburgh, PA, USA

**Keywords:** emotion, cognition, cognitive control, development, adolescence, puberty, affective disorders

## Abstract

Evidence from longitudinal studies suggests that adolescence may represent a period of vulnerability that, in the context of adverse events, could contribute to developmental trajectories toward behavioral and emotional health problems, including affective disorders. Adolescence is also a sensitive period for the development of neural systems supporting cognitive-affective processes, which have been implicated in the pathophysiology of affective disorders such as anxiety and mood disorders. In particular, the onset of puberty brings about a cascade of physical, hormonal, psychological, and social changes that contribute in complex ways to the development of these systems. This article provides a brief overview of neuroimaging research pertaining to the development of cognitive-affective processes in adolescence. It also includes a brief review of evidence from animal and human neuroimaging studies suggesting that sex steroids influence the connectivity between prefrontal cortical and subcortical limbic regions in ways that contribute to increased reactivity to emotionally salient stimuli. We integrate these findings in the context of a developmental affective neuroscience framework suggesting that the impact of rising levels of sex steroids during puberty on fronto-limbic connectivity may be even greater in the context of protracted development of prefrontal cortical regions in adolescence. We conclude by discussing the implications of these findings for future research aimed at identifying neurodevelopmental markers of risk for future onset of affective disorders.

## Introduction

A plethora of clinical and epidemiological studies examining developmental trajectories toward psychopathology have identified adolescence as a potential window of vulnerability (Costello et al., 1996, 2011; Pine et al., 1998; Merikangas et al., 2007). Early adolescence, with the onset of puberty, represents a particularly vulnerable developmental period for the onset of behavioral and emotional health problems. Indeed, although adolescence represents one of the healthiest periods of the life span with respect to physical health, paradoxically, there is mounting evidence suggesting that overall morbidity and mortality rates increase 200–300% (Ozer et al., 2002). For instance, in addition to the increase in the prevalence for mood disorders and substance use disorders, the rate of accidents, suicide, alcohol and substance use, eating disorders, HIV, unwanted pregnancies all increase drastically during this developmental period (Force, 1996; Ozer et al., 2002). The sources of this drastic increase in the rates of adolescent death and disability appear to be primarily related to problems with emotion regulation.

Research in the fields of developmental cognitive and affective neuroscience has blossomed over the past decade allowing researchers to generate neural models that could help elucidate some of the potential neurodevelopmental mechanisms underlying vulnerability for emotion dysregulation in adolescence and risk for psychopathology such as affective disorders and substance abuse disorders (Nelson et al., 2005; Ernst et al., 2006; Steinberg, 2007; Blakemore, 2008; Casey et al., 2010). Some of these models have emphasized adolescent social reorientation toward peers (e.g., Nelson et al., 2005), changes in social cognition (Blakemore, 2008), reward processing (Bjork et al., 2004), and the balance and/or integration of emotional reactivity (i.e., threat, reward) and cognitive control (Steinberg, 2005, 2007; Casey et al., 2010). Despite some points of divergence across these various models, there is agreement that the onset of puberty is associated with increased reactivity in subcortical regions to emotionally salient information (e.g., amygdala, ventral striatum) in ways that create new challenges for cognitive control processes supported by prefrontal systems that mature later in adolescence. Such a maturational gap is therefore thought to contribute to increased vulnerability for emotion dysregulation and possibly the onset of psychopathology such as affective disorders in at-risk youth. Indeed, there is mounting evidence demonstrating the influence of sex hormones on the regulation of emotional responses and affective states in adults (Van Wingen et al., 2010, 2011; Volman et al., 2011) along with recent findings suggesting that sex hormones impact adolescent brain development (for a review, see Blakemore et al., 2010; Peper et al., 2011; Ladouceur et al., 2012). Furthermore, there is growing evidence that puberty is more associated to the onset of depression than age (Angold et al., 1999; Joinson et al., 2012). For instance, a recent population-based study investigating a wide range of influences on the health and development of children reported an association between depression symptoms and pubertal maturation in girls (i.e., breast development, which is linked with a rise in estrogen (Joinson et al., 2012). It is therefore reasonable to conceive that increased risk for affective disorders in adolescence may be mediated by altered puberty-specific changes in the functioning of fronto-limbic systems that support cognitive control-emotion interactions underlying emotion regulatory processes.

The current review will focus particularly on adolescent development of fronto-limbic systems that support processes at the interface of cognitive control and emotion as a way to elucidate potential neurodevelopmental mechanisms underlying vulnerability for emotion dysregulation and onset of affective disorders in at-risk youth. In particular, the focus will be on cognitive control-emotion interactions implicated in the modulation of cognitive resources in the context of emotionally salient information in view of generating an appropriate response as this would appear to be of particular relevance for emotion dysregulation and its consequences in adolescence. As such, the review will feature particular studies that document developmental changes in the influences of emotionally salient cues (e.g., threat-related or appetitive) on cognitive control processes such as attention, working memory, and response inhibition, given that dysfunction in these areas have been implicated in affective disorders (Phillips et al., 2003, 2008). The review will not include findings related to cognitive control processes having emotional stimuli as the object of cognition (e.g., maintaining emotional stimuli in working memory) or processes that involve emotional decision making (i.e., risk taking). Though these are important areas of study, particularly with regard to adolescent behavioral and emotional health, they involve various networks beyond those involved in the cognitive modulation of emotional distracters. Furthermore, it is important to note that there have been recent reviews on the neural substrates of cognition-emotion interactions in normative samples (Elliott et al., 2010; Dolcos et al., 2011), adult mood disorders (Elliott et al., 2010), and development (Mueller, 2011). Consequently, the current review will focus primarily on evidence from animal studies and human neuroimaging studies documenting puberty-specific influences on fronto-limbic systems and how such evidence may enhance our understanding of the development of cognitive-affective processes in adolescence. Finally, I will discuss these findings with regard to their implications for contributing to the vulnerability of emotion dysregulation and potentially the onset of affective disorders in at-risk youth.

## Studies of cognitive-affective processes in adolescence

Using a paradigm that more or less constrains attention to the emotional features of facial expressions, Monk et al. (2003) reported that adolescents relative to adults showed greater activation in the anterior cingulate, bilateral orbitofrontal cortex, and right amygdala in response to the fearful relative to neutral faces (Monk et al., 2003). This was one of the first fMRI studies to examine selective attention to emotional and nonemotional features of stimuli in adolescence. These findings suggest that developmental changes in attentional capacities may play an important role in the modulation of attention to emotionally salient information. Using the same task in adolescents diagnosed with generalized anxiety disorder, Mcclure et al. (2007) reported that while attending to their own subjective fear, anxious youth, but not healthy controls, exhibited greater amygdala, ventral prefrontal cortex, and anterior cingulate cortex to fearful vs. happy faces (Mcclure et al., 2007). Such findings suggest that anxious adolescents may exhibit altered functioning of threat systems in the context of subjective fearful experiences.

In a developmental study, Tottenham et al. (2011) examined the performance of children (5–12 years old), adolescents (13–18 years old), and adults (19–28 years old) using a block design emotional go/no-go task. In this study, participants were instructed to press a button (go) to particular emotional faces and to not press the button (no-go) to other emotional faces (Tottenham et al., 2011). Findings indicated that performance on the task improved with age. Nevertheless, across the age groups, false alarms occurred more frequently to emotional face no-go stimuli relative to neutral face no-go stimuli, which were interpreted as indexing reduced inhibitory control in the context of emotionally salient emotional information. Using a similar version of the task in the fMRI scanner, Hare et al. (2008) documented elevated amygdala activation in adolescents relative to children and adults on this task (Hare et al., 2008). Furthermore, elevated amygdala and reduced ventral prefrontal cortical activation positively correlated with slower reaction times to the fearful (vs. happy) face target stimuli. Functional connectivity analyzes revealed that strength of VLPFC-amygdala coupling was correlated with greater habituation of amygdala activity to fearful face targets in adolescence (Hare et al., 2008).

Using a visual n-back working memory task with emotional face distracters (i.e., Emotional Face n-back (EFNBACK) task), we examined performance on trials with emotional (i.e., happy and fearful face) distracters and varying in working memory load (i.e., 2-back versus 0-back condition) in a normative sample of children (8–10 years old), young adolescents (mean age = 11–13 years), older adolescents (14–17 years), and adults (18–27 years). Results indicated that youth high in trait anxiety exhibited slower reaction times to fearful face distracters in the 2-back condition and that such an effect was greater in children than adolescents (Ladouceur et al., 2009). Such findings are consistent with previous behavioral data on a similar emotional working memory task in a clinical sample of children and adolescents diagnosed with anxiety, depression, and comorbid anxiety and depression (Ladouceur et al., 2005). In that study, we showed that relative to healthy controls, youth with depression and comorbid anxiety and depression had significantly slower reaction times on negative emotional backgrounds compared to neutral backgrounds. In contrast, youth in the healthy low-risk group showed slower reaction times to the positive emotional background, suggesting an attentional bias to positively valenced stimuli; such an effect was not present in the groups with affective disorders (Ladouceur et al., 2005). Recent neuroimaging studies using cognitive-affective tasks with fMRI in youth with anxiety and mood disorders have reported alterations in the functioning of VLPFC and amygdala, suggesting that VLPFC modulation of amygdala may contribute to affective biases reported in these clinical populations (Monk et al., 2008; Pavuluri et al., 2008). Together, these findings suggest that using cognitive-affective tasks such as the emotional working memory paradigm enables researchers to investigate the development of attentional control processes (implicated in resisting interference from emotionally salient distracters) and examine to what extent the development of the neural systems that support these processes may be altered in youth at risk for or diagnosed with affective disorders.

Some researchers have also examined the influence of incentives on cognitive processes. For instance, using an emotional antisaccade task, Geier and colleagues reported differences in performance and associated neural activation in fronto-striatal regions in adolescents relative to adults (Geier et al., 2010). An antisaccade is an eye movement to the opposite direction of a suddenly appearing target. This movement requires inhibiting a prepotent response to the target, and the initiation of an alternate goal-relevant response in the opposite direction signaled by the sudden onset of the stimulus. Geier and colleagues examined the effects of reward on antisaccades, during the cue stage, saccade preparation stage, and saccade execution stage. In order to assess neural responses across these three stages, a time-course analysis over 18 s post-trial onset was employed. They reported greater ventral striatum activity to the saccade preparation in adolescents than adults but during the incentive cue, it was recruited more strongly in adults than adolescents; there were no group differences in the saccade execution stage. Neural regions commonly recruited during antisaccade were generally less activated in adolescents than adults in response to neutral trials and showed no age-group differences to reward trials. Interestingly, however, this study demonstrated that adolescents tended to exhibit greater recruitment of reward circuitry during saccade preparation to incentive trials. These findings are consistent with those of another recent study in which adolescents exhibited greater activation in reward circuitry and prefrontal regions than adults on a continuous performance task (CPT) (Smith et al., 2011). In this study, Smith and colleagues compared, in adolescents relative to adults, behavioral performance and neural activity on three types of trials (non-targets, rewarded targets, and on-rewarded targets) of CPT. Findings from behavioral data analyzes revealed that adolescents responded significantly faster to rewarded vs. non-rewarded targets whereas no such differences were observed in adults. These behavioral findings suggest that being rewarded had significant impact on sustained attention in adolescents than adults. Moreover, findings from fMRI analyses revealed a significant positive correlation between age and neural activity to rewarded (vs. non-rewarded) targets in neural regions implicated in sustained attention (e.g., dorsolateral prefrontal cortex, ventromedial orbital frontal cortex), suggesting that age modulated the effects of reward on activity in neural regions implicated in sustained attention.

In sum, these findings suggest that cognitive control may be more challenged in the face of emotionally salient or incentive-laden distracters in adolescents relative to adults. We acknowledge that our review of cognitive-affective findings in adolescence was not exhaustive. The purpose was not to provide an exhaustive literature review since this has been accomplished elsewhere (e.g., Mueller, 2011). Rather the aim was to provide some examples of research findings demonstrating that the influence of emotionally salient information on the functioning of neural systems supporting cognitive processes including cognitive control undergo important neuro-maturational changes in adolescence. In this review, I will demonstrate that the onset of puberty is associated with increased reactivity to emotionally salient information in ways that create challenges for cognitive control systems. Furthermore, these pubertal influences on fronto-limbic systems may be associated with reduced modulation of attention in the context of emotional distracters and increased vulnerability to emotion dysregulation. Such a framework complements existing neurobiological models of adolescent brain development (Casey et al., 2010; Ernst et al., 2011) but attempts to move beyond age-related effects to focus more specifically on neurodevelopmental changes occurring at puberty and how such changes may help explain increases in the escalating rates of adolescent death and disability related to problems of emotion regulation (e.g., mood disorders, suicide, accidents, etc.).

## What developmental factors might influence cognitive-affective processes?

### Rise in sex hormones during puberty

Puberty refers to a specific set of processes implicating changes in physical and reproductive maturation. Although the majority of these changes occur early to mid-adolescence, as described below, some (e.g., adrenarche and luteinizing hormone secretion), however, can start in childhood. As such, puberty is often considered as the beginning of adolescence, a developmental period between childhood and adulthood that encompasses changes at multiple levels. This transitional developmental period not only implicates changes associated with pubertal maturation but also changes in physical growth, psychological functioning, and social experiences (Dahl and Spear, 2004; Dorn et al., 2006).

Puberty includes important changes in the functioning of the neuroendocrine system (for a review, see Dorn et al., 2006; Natsuaki et al., 2009; Blakemore et al., 2010). The earliest phase of puberty or “prepuberty,” which begins between 6–9 years old in girls and about 1 year later in boys (Cutler et al., 1990; Parker, 1991), involves the rising of androgens that are secreted by the adrenal glands. These include dehydroepiandrosterone (DHEA), its sulfate (DHEAS), and androstendione (Grumbach and Styne, 2003). The rising of these hormones refers to what is known as the beginning of *adrenarche*. The maturation of primary sexual characteristics (i.e., ovaries and testes) and the full development of secondary sexual characteristics (i.e., pubic hair, breast, and genital development) is associated with the activation of the hypo-thalamic-pituitary gonadal (HPG) axis (Demir et al., 1996; Delemarre-Van De Waal, 2002). The rising of these sexual hormones represents a second phase of puberty known as *gonadarche*, which begins at about 9–10 years old in girls and approximately 1–2 years later in boys (Marshall and Tanner, 1969, 1970). This pubertal period includes the onset of menses, or *menarche*, in girls, and the onset of nocturnal emission, or *spermarche*, in boys. Menarche in girls tends to be an event that occurs rather late in the pubertal process. Spermarche in boys represents the transition from prepubertal to pubertal, and occurs on average at approximately 13–14 years old (Marshall and Tanner, 1969, 1970). The development of secondary sexual characteristics occurs gradually and as such, has been organized into stages (e.g., Tanner stages), which has allowed clinicians and researchers to assess variations in pubertal maturation.

#### Individual variations in pubertal maturation processes

Most adolescents will exhibit the above-described endocrinological changes associated with pubertal maturation but not in a uniform manner as there is considerable individual variation in both the “timing” and “tempo” of puberty (Styne and Grumbach, 2002; Dorn et al., 2006). Variation in the timing refers to the level of pubertal maturation relative to same-age peers and variation in the tempo refers to the adolescent's rate of intra-individual change in pubertal status (i.e., the amount of time brains are exposed to sex hormones). Because pubertal maturation is a dynamic biological process, variations in this process may be influenced by multiple developmental factors some of which can precede the onset of puberty *per se* (e.g., nutritional history, stressful life events, and family conflict). Discussion about research findings documenting how each of these factors contributes to variations in pubertal timing and tempo is beyond the scope of this review (for addition information, see Ellis, 2004; Ellis et al., 2011a,b). Nevertheless, there is a general trend toward younger age of puberty onset in the United States—particularly age of breast development and menarche among girls (Biro et al., 2012). Variations in both pubertal timing and pubertal tempo have been related to emotional functioning, particularly in girls (Ellis et al., 2011b). For instance, there is growing evidence that girls who mature earlier than their on-time or later-developing peers are at increased risk for psychopathology, including depression, early initiation of substance use, early sexual initiation and pregnancy and other emotional, and behavioral problems (Dick et al., 2000; Deardorff et al., 2005; Mendle et al., 2010; Joinson et al., 2012). In particular, Joinson et al. (2012) recently reported that more advanced breast development was associated with increased depressive symptoms in adolescence. This was a large multi-wave longitudinal study, which included measures of the different hormonal axes of puberty (i.e., secondary sexual characteristics associated with adrenarche and gonarche) (Joinson et al., 2012). Because breast development is due to a rise in estrogen, these findings support previous data demonstrating that estradiol levels in girls is linked with increased depression in girls (Angold et al., 1999). Fewer studies have reported similar influences in boys. Some have reported, however, that the influence of pubertal timing and tempo on emotional functioning may be mediated by the quality of peer relationships (Mendle et al., 2012). In light of evidence from animal studies showing high densities of gonadal hormone receptors in medial temporal regions implicated in emotion processing (e.g., amygdala, striatum) (Simerly et al., 1990; Sarkey et al., 2008), it is thus reasonable to predict that adolescents who exhibit onset of puberty earlier than their on-time or later developing peers would also exhibit greater reactivity to emotionally salient information.

### Heightened emotional reactivity in the context of protracted development of regulatory control

Prominent models of adolescent brain development highlight a temporal “mismatch” in the development of neural systems supporting emotional reactivity and regulation (Steinberg, 2005; Ernst et al., 2006; Casey et al., 2011). These models propose that adolescence represents a period of increased emotional reactivity during early adolescence along with a more gradual and protracted development of regulatory control. Neural regions that subserve cognitive control functions show age-related functional changes that continue well into late adolescence and early adulthood (Lewis, 1997; Sowell et al., 2003; Luna et al., 2004) and are thought to mature irrespective of pubertal timing (Steinberg, 2005). With the onset of puberty occurring earlier over the past century (Worthman, 1999) and prefrontal systems supporting regulatory control not reaching full maturity until early adulthood, this creates a potential maturational gap creating a possible “imbalance” in fronto-limbic circuitry that may lead to dysregulated behavior and affect, particularly in vulnerable youth. This has led to a metaphor for the historical advancement of puberty as “starting the engines with an unskilled driver” (Dahl and Spear, 2004). This dilemma has been proposed to explain, in part, the fact that adolescents often make poor decisions that lead to negative consequences (e.g., accidents, drug use) despite cognitively understanding the risks involved (Cauffman and Steinberg, 1995; Reyna and Farley, 2006). Although such models could serve to help explain developmental effects observed in adolescence during the performance of cognitive-affective tasks, most of the research thus far has focused on age-related changes in the development of cognitive control processes and we know very little about the influence of pubertal maturation on the development of these systems—particularly as it relates to effects on neural activity in subcortical limbic regions and their modulation by prefrontal cortical circuitry.

### Social reorientation: changes in motivational tendencies

Early adolescence is also characterized by concurrent changes in motivational tendencies geared toward social peer interactions. As discussed in (Forbes and Dahl, 2010) the increase of sex hormones with the onset of puberty plays a crucial role on the re-orientation of social behavior in adolescence (Forbes and Dahl, 2010). Specifically, social affiliation with peers and romantic interests becomes increasingly important thereby eliciting strong emotional reactions (Larson and Asmussen, 1991). Testosterone, for instance, plays an important role in sensation-seeking (Martin et al., 2002), risk taking (Vermeersch et al., 2008), and social dominance (Eisenegger et al., 2011). Consequently, the saliency of social cues becomes reconfigured according to expectations of potential gain or loss in social status or affiliation. Some have argued that neural regions involved in processing motivationally relevant information undergo remodeling during adolescence (Spear, 2000). Such remodeling has been observed in non-human species and is believed to have adaptive value in preparing the individual to survive away from parental caretakers by encouraging the adolescent to develop new bonds and explore novel areas (Spear, 2000). These changes in motivational tendencies would therefore promote a natural tendency to explore new things and take more risks—particularly when these have social salience (e.g., gain attention and/or admiration from peers). Such social reorientation in adolescence would therefore magnify the emotional saliency of social cues in ways that would impact the functioning of fronto-limbic circuitry that support cognitive-affective processes.

## Sex steroids modulate activity in and connectivity between neural regions supporting cognitive-affective processes

As discussed previously, puberty is associated with significant endocrinological changes, such as a vast increase in the sex steroids testosterone and estradiol released from the gonads. Findings from animal studies indicate that sex steroids such as testosterone, estradiol, and their antecedents (DHEA) impact the organization of neural circuits that support social, sexual and emotional behavior (Sisk and Zehr, 2005; Ahmed et al., 2008; Schulz et al., 2009). These effects may be mediated by the influence of sex hormones on fronto-striatal-limbic systems given the high densities of steroid hormone receptors in medial temporal regions (Simerly et al., 1990; Sarkey et al., 2008), such as striatum, amygdala, and hippocampus, as well as the prefrontal cortex (Pfaff and Keiner, 1973; Simerly et al., 1990). As reviewed in Eisenegger et al. (2011) and Bos et al. (2011), testosterone is produced in both boys and girls and is viewed as a “social hormone” because of its role in promoting the search for, and maintenance of, social status and alterations in the appraisal of threats and rewards—particularly when these are relevant to social status (Bos et al., 2011; Eisenegger et al., 2011). Estradiol, a metabolite of testosterone that is critical for female reproductive function, has been linked to emotional behavior (Walf and Frye, 2006) and to various cognitive functions (Berman et al., 1997; Jacobs and D'esposito, 2011).

**Figure 1:**
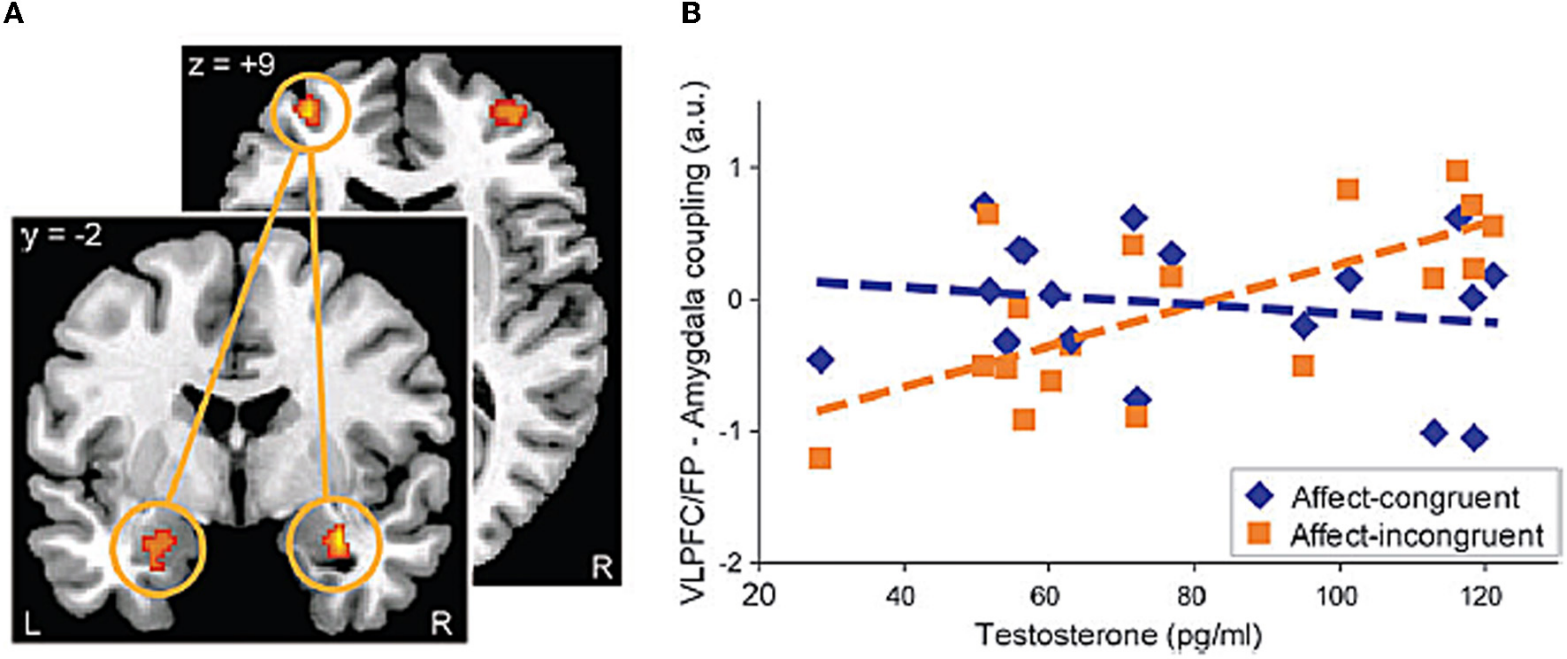
**Example of the effects of testosterone on the functional connectivity between prefrontal cortical regions (i.e., VLPFC/FP) and subcortical regions (i.e., amygdala) during a task that recruits cognitive-affective processes. (A)** Coupling between left VLPFC/FP and right amygdala, which was significantly (FWE, *p* < 0.05) modulated by testosterone during the affect-incongruent compared to the affect congruent condition of the Approach-Avoid task. **(B)** Scatter plot visualizing the positive correlation between testosterone and the VLPFC/FP-amygdala connectivity for the affect-incongruent versus the affect congruent trials. (Reprinted with permission from Cerebral Cortex, Oxford Publishers).

Recent neuroimaging studies examining the influence of sex steroids on fronto-limbic systems in adults provide important clues about potential mechanisms regarding the influence of puberty-related neuroendocrine changes on fronto-limbic function. The majority of the studies have focused on individual differences in testosterone levels and few studies have examined the effects of estradiol (for a review see, Walf and Frye, 2006), with the exception of work in post-menopausal women (Naftolin and Malaspina, 2007). For instance, a number of studies have demonstrated that testosterone influences neural activity in amygdala and regions of the prefrontal cortex (Stanton et al., 2009; Manuck et al., 2010; Mehta and Beer, 2010; Van Wingen et al., 2010). Specifically, amygdala response to emotional faces (i.e., fearful/angry) was associated with higher levels of serum testosterone concentrations in young men (Derntl et al., 2009; Manuck et al., 2010) and women (Hermans et al., 2008; Derntl et al., 2009; Van Wingen et al., 2010). A potential mechanism underlying these changes in amygdala responses might be that testosterone influences fronto-limbic connectivity implicated in emotion regulation. Indeed, Van Wingen et al. (2010) demonstrated that testosterone induces a “functional decoupling” between amygdala and ventral prefrontal cortex (i.e., BA47) activity, which they interpreted in terms of reduced (automatic) regulatory influence of the prefrontal regions over the amygdala (Van Wingen et al., 2010). A more recent study employed a cognitive-affective task, the Approach-Avoid Task, which was designed to assess implicit approach-avoidance action tendencies by manipulating the congruency of the stimuli to which participants respond (Volman et al., 2011). In that study, young male participants were asked to use a joystick to respond to visually presented emotional facial expressions (happy, anger, neutral), which are known to produce automatic approach (happy) and avoidance (angry) tendencies. The neutral face served as the control condition. They were asked to either pull the joystick toward themselves (approach) or push it away from themselves (avoid) based on the valence of the emotional facial expressions. Volman et al. (2011) examined the influence of endogenous hormones on the functional connectivity between VLPFC and amygdala, based on evidence that such regions are implicated in the (voluntary) regulation of prepotent emotional action tendencies. As depicted in Figure 1, results demonstrated that endogenous testosterone modulated effective connectivity between VLPFC and frontal pole (FP) and the amygdala in the affect-incongruent condition reflecting regulation of emotional action tendencies (Volman et al., 2011). These findings suggest that high levels of endogenous testosterone in healthy males are associated with less recruitment of VLPFC/FP to modulate amygdala activity. Although these findings are intriguing, the specific neurochemical mechanism by which testosterone could influence fronto-limbic systems remains uncertain (e.g., perhaps mediated in part through its conversion to other streroids) (Bodo and Rissman, 2006). Nevertheless, these findings suggest that sex hormones (i.e., testosterone) appear to influence not only activity in but also connectivity between neural regions implicated in emotion regulation.

## Puberty-related changes in adolescent brain development

The above findings provide compelling evidence for the direct influence of sex hormones on brain function. As such, given the increase of sex hormones that occurs with the onset of puberty (and during prepuberty), it is reasonable to expect that pubertal maturation may exert direct and indirect influences on neurodevelopment. We next briefly review recent findings that demonstrate how changes in pubertal maturation may influence changes in the functioning of neural systems implicated in emotion processing and regulation.

### Structural neuroimaging studies

A number of studies have demonstrated the influence of pubertal maturation on gray matter development (Giedd et al., 2006; Peper et al., 2009; Bramen et al., 2010, 2012) and more recently white matter development (Asato et al., 2010; Herting et al., 2011; Peper et al., 2011; Ladouceur et al., 2012). Gray matter is composed of cell bodies, dendrites, and nonmyelinated axons of neurons, including glial cells and capillaries. Cross-sectional and longitudinal studies have documented non-linear age-related changes in gray matter volume, density, and thickness (mostly cortical areas), which would appear to follow an inverted-U shape reaching a peak in adolescence (Giedd et al., 1999; Sowell et al., 2003). White matter provides the structural architectural organization of the brain. It is made up primarily of myelinated axons that serve to facilitate communication between neural regions creating neural networks, which subserve complex cognitive and affective functions (Paus, 2010). Several MRI studies have documented linear age-related increases in white matter volume between childhood and adolescence, reaching a plateau in adulthood (Giedd et al., 1999).

Numerous studies have reported sex differences in the development of gray and white matter during adolescence (for a review, see Lenroot and Giedd, 2010). Given that puberty typically onsets later in males than females, such findings provide some initial clues with regard to the potential influences of pubertal maturation on brain development (i.e., sexual dimorphism). That is, even though certain MRI studies do not include direct measures of pubertal maturation (e.g., hormone levels, self-report), findings indicating significant sex differences and sex by age interactions could be interpreted in terms of hormonal influences on gray and white matter development or onset of puberty. Although these findings are compelling, they are beyond the scope of the current review and have been described in previous reviews (e.g., Giedd et al., 2006; Lenroot et al., 2007; Lenroot and Giedd, 2010; Ladouceur et al., 2012). Indeed, a more direct approach is to include measures of pubertal maturation such as self-report, physical exams, or saliva or blood essays of reproductive hormones. Another important methodological consideration that few studies have incorporated is the recruitment of males and females according to the approximate age of puberty onset (i.e., gonarche), which is begins at about 9–10 years old in girls (Marshall and Tanner, 1969) and approximately 1 year later in boys (Marshall and Tanner, 1970).

Recent research studies have begun to document the role of pubertal maturation on the developing brain (Perrin et al., 2008; Blakemore et al., 2010; Bramen et al., 2010, 2012; Peper et al., 2011). Such studies reveal that sex hormones exert unique influences on gray matter volume (Neufang et al., 2009; Bramen et al., 2010), density (Peper et al., 2009), and thickness (Raznahan et al., 2010; Bramen et al., 2012). A few recent studies have reported associations between pubertal maturation, changes in sex hormones, and white matter development (Peper et al., 2011; Ladouceur et al., 2012). Such changes included, for instance, associations between testosterone levels in boys and increases in white matter volume (Perrin et al., 2008) and increased integrity of white matter tracts connecting frontal and temporal regions as well as frontal and subcortical regions with more advanced pubertal status (based on Tanner stage assessment) (Asato et al., 2010). Interestingly, there is also evidence for associations between increasing level of relationship between levels of luteinizing hormone (LH) in 9-year old twins, which is considered as one of the first endocrinological markers of puberty in both boys and girls, and larger white matter density (Peper et al., 2008). Furthermore, there is evidence that the androgen receptor gene, whose length modulates the action of the androgen receptor, would appear to play an important role in regulating gray matter (Raznahan et al., 2010) and white matter development (Perrin et al., 2008; Paus, 2010). Given the relatively small number of studies that have examined specifically associations between pubertal maturation and brain structure development, findings thus far indicate a significant contribution of puberty, in particular testosterone in boys and its precursor LH (in both sexes), on the development of frontal and temporal regions (and their structural connections), which may have implications for the development of cognitive-affective processes supporting emotion processing and regulation in adolescence.

### Functional neuroimaging studies

To date, only a handful of studies have investigated effects of puberty and sex hormones using functional neuroimaging. Before describing these findings, it is important to first note that the use of appropriate methodological designs is critical in order to examine specific pubertal influences on the functioning of fronto-limbic systems. To what extent are studies able to disentangle age effects from pubertal maturation is key to addressing puberty-specific developmental questions since age and puberty are closely correlated with each other (and chronological age is measured with a much greater precision than categories for pubertal stage) (Shirtcliff et al., 2009; Spear, 2010). Given that boys typically reach puberty later than girls, it is important, for instance, to recruit participants within a narrow age-range to account for potential age-related effects but also to take into account sex differences in pubertal maturation (Dorn et al., 2006; Shirtcliff et al., 2009). Such methodological designs have inherent constraints with regard to subject recruitment, which might explain the paucity of research in this area. Nevertheless, there is an emerging literature that demonstrates the feasibility of using such designs in order to address these important developmental questions. Many other factors are important to consider and these have been reviewed elsewhere (Dorn et al., 2006; Shirtcliff et al., 2009).

Using such methodological approaches our group and others have examined the influences of puberty on psychophysiological and neural indices of emotional reactivity during emotion processing and regulation tasks. For instance, Silk et al. (2009) examined associations between pubertal maturation and physiological and subjective reactivity to emotional words (Silk et al., 2009). In this study, pupil dilation was assessed among 32 pre-/early pubertal and 34 mid-/late pubertal typically developing children and adolescents while they an emotional word valence identification paradigm with positive, negative, and neutral words. Pupil dilation, which is a peripheral index of brain activation, is generally greater in response to stimuli that require greater cognitive load or that have greater emotional intensity (Siegle et al., 2004). Our findings showed that mid-/late pubertal youth had greater peak pupil dilation to affective words than pre-/early pubertal youth, even controling for participants' age. This peak pupil dilation was correlated with heightened memory for emotional but not neutral words on an unexpected free recall task, suggesting that such peak pupil dilation measure may be linked to emotionally-relevant processing. These findings are consistent recent associations between pubertal maturation and neural activity to emotional facial expressions (Moore III et al., 2012). There is also evidence that adolescent-levels of testosterone correlates with maturational changes in neural systems supporting reward processing in adolescents (Forbes and Dahl, 2010; Op De Macks et al., 2011). Furthermore, threat-related reactivity to social cues has been associated with levels of depression symptoms in peri-pubertal adolescents (Forbes et al., 2011). Taken together, these findings of pubertal changes in the functioning of fronto-limbic circuitry implicated in emotion processing and regulation has tremendous implications for understanding potential developmental mechanisms implicated in risk for affective disorders in adolescence.

## Conclusion and future directions

**Figure 2:**
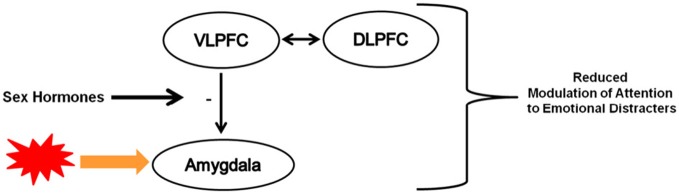
**Simplified illustration of heuristic model describing the potential influence of the increase in sex hormones during pubertal maturation on the functioning of fronto-limbic circuitry implicated in the modulation of attention to emotionally salient distracters.** For simplicity, involvement of other neural regions and their interactions with these and other regions were omitted. (+): positive modulation, (−) negative modulation, thickness of arrows reflects relative weight of impact. VLPFC: ventrolateral prefrontal cortex; DLPFC: dorsolateral prefrontal cortex.

The goal of this review was to briefly synthesize research findings demonstrating developmental changes in cognitive-affective processes and underlying fronto-limbic circuitry. The second and perhaps more important goal was to highlight evidence that speaks to the potential influence of puberty on the development of this circuitry in ways that could have implications for understanding risk for affective disorders. Notwithstanding the importance of age-related neuromaturational changes to the functioning of this circuitry (Mueller, 2011; Tottenham et al., 2011), very little attention has been dedicated to the potential impact of pubertal maturation on the reactivity of subcortical regions (e.g., amygdala, ventral striatum) and how such heightened emotional reactivity early in adolescence may create new challenges for later developing cognitive control and underlying neural systems. As illustrated in Figure 2, I propose that with the increase in sex hormones during puberty, adolescents may exhibit reduced modulation of attention to emotionally salient distracters because of the potential influence of sex hormones on the functional connectivity between prefrontal cortical regions implicated in the modulation of subcortical regions. The extent to which puberty-related changes in circulating levels of sex hormones impacts cognitive-affective interactions remains largely unexplored. To date, the emerging literature has focused on emotion information processing (e.g., Moore III et al., 2012) or reward processing (e.g., Forbes et al., 2011) and few studies have used tasks that recruit higher-order cognitive control processes. However, findings from Hare et al. (2008) showing that the strength of VLPFC-amygdala coupling was correlated with greater habituation of amygdala activity to fearful face targets in adolescence during an emotional go-nogo task provide some interesting clues about the potential effects of pubertal maturation on these systems (Hare et al., 2008). Future research employing appropriate methodological designs are needed to determine whether these findings are indeed age- versus puberty-related effects.

In addition to pubertal changes in the functioning of these fronto-limbic systems, there are also notable puberty-related changes in motivational tendencies that become increasingly oriented toward peer social contexts (Nelson et al., 2005). This new set of motivational tendencies brings about a reconfiguration in the saliency of emotional and social cues because of their prediction of potential changes in social status/evaluation (e.g., heightened reactivity of threat system to emotional facial expressions that could be linked to worries of being rejected by unfamiliar peers) (Forbes and Dahl, 2010; Silk et al., 2012a). Some have demonstrated that these social information processing systems become more sensitive or reactive during puberty, particularly to cues of social threat or reward (Dahl, 2001; Nelson et al., 2005; Steinberg, 2007; Spear, 2010). For example, Silk et al. (2012a,b) recently reported increased sensitivity to social evaluation across adolescence in a virtual peer interaction chatroom task. Specifically, pupillary reactivity to peer rejection and visual biases toward acceptance stimuli increased linearly with age among 9- to 17-year-olds (Silk et al., 2012b). Therefore, it is possible that the social nature of emotional distracters may require greater modulation of attention and recruitment of modulatory prefrontal cortical systems because of the heightened motivational saliency of such stimuli.

Another factor to consider is the individual variation in pubertal maturation processes and how these might contribute to increased vulnerability to emotion dysregulation and possibly the onset of psychopathology such as affective disorders in at-risk youth. The general trend toward earlier onset of puberty, particularly in girls, may be associated with greater reactivity in subcortical regions. Such reactivity may be enhanced in the context of threat to social status (i.e., being rejected by a peer), stressful life events, and in youth having a family history of affective disorders. Such heightened reactivity in young at-risk youth in the context of protracted development of prefrontal cortical systems supporting regulatory processes and potentially reduced fronto-limbic connectivity associated with changes in the level of hormones could contribute to developmental trajectories toward affective disorders. Of note, there is evidence showing that stressful family environment (i.e., family conflict) influences pubertal timing and tempo (Ellis, 2004) and that this effect might be greater in a subset of youth who are particularly biologically sensitive to social context (Ellis et al., 2011b). Future research is therefore needed to investigate the specific influence of pubertal maturation on fronto-limbic function supporting cognitive control-emotion interaction in normally developing adolescents and at-risk youth in order to test the above hypotheses and elucidate potential mechanisms for the increased risk of affective disorders in adolescence, particularly in girls.

In sum, there is an emerging literature suggesting that pubertal maturation may exert specific influences on adolescent brain development (e.g., Silk et al., 2009; Blakemore et al., 2010; Forbes et al., 2011; Op De Macks et al., 2011; Peper et al., 2011; Ladouceur et al., 2012; Moore III et al., 2012). Such influences may have greatest impact on cognitive-affective processes and as such, potentially alterations in emotion regulation in vulnerable youth. Animal studies indicate that sex hormones impact dopamine function (e.g., Aubele and Kritzer, 2011). It is therefore possible that the impact of sex hormones on fronto-limbic function may be mediated by their impact on neurotransmitter systems such as dopamine—which undergoes important maturational changes in adolescence (Wahlstrom et al., 2010; Ernst et al., 2011). Given the role of dopamine in modulating cognitive control processes (Jacobs and D'esposito, 2011), one avenue of research might be to examine the role of dopamine and associations with functioning of neural systems supporting processes at the interface of cognition and emotion. Furthermore, evidence from structural as well as functional neuroimaging studies demonstrate that fronto-limbic systems are implicated in the pathogenesis of affective disorders, especially ventromedial-amygdala and frontal-thalamic-striatal systems (Mayberg, 2001; Phillips et al., 2008; Savitz and Drevets, 2009; Price and Drevets, 2010). Because rates of depression (Angold et al., 1998, 1999) are more strongly associated with puberty than age, it is possible that puberty-specific influences on the development of these neural systems may render particularly vulnerable youth (i.e., genetic predisposition, early-life stressors) at increased risk for affective disorders. Thus, future longitudinal research studies investigating the neuroendocrine, social, and age-related influences on the maturation of neural systems supporting cognitive-affective processes and associations with emotion regulation processes would contribute to advancing the field of developmental cognitive and affective neuroscience as well as our knowledge about potential neurodevelopmental markers of risk for affective disorders in adolescence.

### Conflict of interest statement

The author declares that the research was conducted in the absence of any commercial or financial relationships that could be construed as a potential conflict of interest.

## References

[B1] Ahmed E. I. Zehr J. L. Schulz K. M. Lorenz B. H. Doncarlos L. L. Sisk C. L. (2008). Pubertal hormones modulate the addition of new cells to sexually dimorphic brain regions. Nat. Neurosci. 11, 995–997 19160494 10.1038/nn.2178 PMC2772186

[B2] Angold A. Costello E. J. Erkanli A. Worthman C. M. (1999). Pubertal changes in hormone levels and depression in girls. Psychol. Med. 29, 1043–1053 10576297 10.1017/s0033291799008946

[B3] Angold A. Costello E. J. Worthman C. M. (1998). Puberty and depression: the roles of age, pubertal status and pubertal timing. Psychol. Med. 28, 51–61 9483683 10.1017/s003329179700593x

[B4] Asato M. R. Terwilliger R. Woo J. Luna B. (2010). White matter development in adolescence: a DTI study. Cereb. Cortex 20, 2122–2131 10.1093/cercor/bhp282 20051363 PMC2923214

[B5] Aubele T. Kritzer M. F. (2011). Gonadectomy and hormone replacement affects *in vivo* basal extracellular dopamine levels in the prefrontal cortex but not motor cortex of adult male rats. Cereb. Cortex 21, 222–232 10.1093/cercor/bhq083 20466748 PMC3025724

[B6] Berman K. F. Schmidt P. J. Rubinow D. R. Danaceau M. A. Van Horn J. D. Esposito G. Osstrem J. L. Weinberger D. R. (1997). Modulation of cognition-specific cortical activity by gonadal steroids: a position-emission tomography study in women. Proc. Natl. Acad. Sci. U.S.A. 94, 8836–8841 9238064 10.1073/pnas.94.16.8836 PMC23156

[B7] Biro F. M. Greenspan L. C. Galvez M. P. (2012). Puberty in girls of the 21st century. J. Pediatr. Adolesc. Gynecol. Available online at http://dx.doi.org/10.1016/j.jpag.2012.05.009 10.1016/j.jpag.2012.05.009 22841372 PMC3613238

[B8] Bjork J. M. Knutson B. Fong G. W. Caggiano D. M. Bennett S. M. Hommer D. W. (2004). Incentive-elicited brain activation in adolescents: similarities and differences from young adults. J. Neurosci. 24, 1793–1802 10.1523/JNEUROSCI.4862-03.2004 14985419 PMC6730402

[B9] Blakemore S. J. (2008). The social brain in adolescence. Nat. Rev. Neurosci. 9, 267–277 10.1038/nrn2353 18354399

[B10] Blakemore S. J. Burnett S. Dahl R. E. (2010). The role of puberty in the developing adolescent brain. Hum. Brain Mapp. 31, 926–933 10.1002/hbm.21052 20496383 PMC3410522

[B11] Bodo C. Rissman E. F. (2006). New roles for estrogen receptor beta in behavior and neuroendocrinology. Front. Neuroendocrinol. 27, 217–232 10.1016/j.yfrne.2006.02.004 16603234

[B12] Bos P. A. Panksepp J. Bluthe R. M. Van Honk J. (2011). Acute effects of steroid hormones and neuropeptides on human social-emotional behavior: a review of single administration studies. Front. Neuroendocrinol. 33, 17–35 10.1016/j.yfrne.2011.01.002 21256859

[B13] Bramen J. E. Hranilovich J. A. Dahl R. E. Chen J. Rosso C. Forbes E. E. Dinov I. D. Worthman C. M. Sowell E. R. (2012). Sex matters during adolescence: testosterone-related cortical thickness maturation differences between boys and girls. PLoS ONE 7:e33850 10.1371/journal.pone.0033850 22479458 PMC3315517

[B14] Bramen J. E. Hranilovich J. A. Dahl R. E. Forbes E. E. Chen J. Toga A. W. Dinov I. D. Worthman C. M. Sowell E. R. (2010). Puberty influences medial temporal lobe and cortical gray matter maturation differently in boys than girls matched for sexual maturity. Cereb. Cortex 21, 636–646 10.1093/cercor/bhq137 20713504 PMC3041011

[B15] Casey B. J. Duhoux S. Cohen M. M. (2010). Adolescence: what do transmission, transition, and translation have to do with it? Neuron 67, 749–760 10.1016/j.neuron.2010.08.033 20826307 PMC3014527

[B16] Casey B. J. Jones R. M. Somerville L. H. (2011). Braking and accelerating of the adolescent brain. J. Res. Adolesc. 21, 21–33 21475613 10.1111/j.1532-7795.2010.00712.x PMC3070306

[B17] Cauffman E. Steinberg L. (1995). The cognitive and affective influences on adolescent decision-making. Temple Law Rev. 68, 1763–1789

[B18] Costello E. Angold A. Burns B. Erkanli A. Stangl D. Tweed D. (1996). The great smoky mountains study of youth. Functional impairment and serious emotional disturbance. Arch. Gen. Psychiatry 53, 1137–1143 8956680 10.1001/archpsyc.1996.01830120077013

[B19] Costello E. Copeland W. Angold A. (2011). Trends in psychopathology across adolescent years: what changes when children become adolescents, and when adolescents become adults? J. Child Psychol. Psychiatry 52, 1015–1025 10.1111/j.1469-7610.2011.02446.x 21815892 PMC3204367

[B20] Cutler G. B. Schiebinger R. J. Albertson B. D. Cassorla F. G. Chrousos G. P. Comite F. A.l. E. (1990). Adrenarche (human, and animals), in Control of the Onset of Puberty, eds Grumbach M. M. Sizonenko P. C. Aubert M. L. (Baltimore, MD: Williams and Wilkins), 506–533

[B21] Dahl R. E. (2001). Affect regulation, brain development, and behavioral/emotional health in adolescence. CNS Spectr. 6, 60–72 17008832 10.1017/s1092852900022884

[B22] Dahl R. E. Spear L. (2004). Adolescent brain development: vulnerabilities and opportunities. Ann. N.Y. Acad. Sci. 1021, 472 10.1196/annals.1308.001 15251869

[B23] Deardorff J. Gonzales N. A. Christopher F. S. Roosa M. W. Millsap R. (2005). Early puberty and adolescent pregnancy: the influence of alcohol use. Pediatrics 116, 1451–1456 10.1542/peds.2005-0542 16322170

[B24] Delemarre-Van De Waal H. A. (2002). Regulation of puberty. Best Pract. Res. Clin. Endocrinol. Metab. 16, 1–12 10.1053/beem.2001.0176 11987894

[B25] Demir A. Voutilainen R. Juul A. Dunkel L. Alfthan H. Skakkebaek N. E. Stenman U. H. (1996). Increase in the first morning voided urinary luteinizing hormone levels precedes the physical onset of puberty. J. Clin. Endocrinol. Metab. 81, 2963–2967 10.1210/jc.81.8.2963 8768859

[B26] Derntl B. Windischberger C. Robinson S. Kryspin-Exner I. Gur R. C. Moser E. Habel U. (2009). Amygdala activity to fear and anger in healthy young men is associated with testosterone. Psychoneuroendocrinology 34, 687–693 10.1016/j.psyneuen.2008.11.007 19136216

[B27] Dick D. M. Rose R. J. Viken R. J. Kaprio J. (2000). Pubertal timing and substance use: associations between and within families across adolescence. Dev. Psychol. 36, 180–189 10749075

[B28] Dolcos F. Iordan A. D. Dolcos S. (2011). Neural correlates of emotion–cognition interactions: a review of evidence from brain imaging investigations. J. Cogn. Psychol. 23, 669–694 10.1080/20445911.2011.594433 PMC3206704 22059115

[B29] Dorn L. D. Dahl R. E. Woodward H. R. Biro F. (2006). Defining boundaries of early adolescence: a user's guide to assessing pubertal status and pubertal timing in research with adolescents. Appl. Dev. Sci. 10, 30–56

[B30] Eisenegger C. Haushofer J. Fehr E. (2011). The role of testoterone in social interaction. Trends Cogn. Sci. 15, 263–271 10.1016/j.tics.2011.04.008 21616702

[B31] Elliott R. Zahn R. Deakin J. F. Anderson I. M. (2010). Affective cognition and its disruption in mood disorders. Neuropsychopharmacology 36, 153–182 10.1038/npp.2010.77 20571485 PMC3055516

[B32] Ellis B. J. (2004). Timing of pubertal maturation in girls: an integrated life history approach. Psychol. Bull. 130, 920–958 10.1037/0033-2909.130.6.920 15535743

[B33] Ellis B. J. Boyce W. T. Belsky J. Bakermans-Kranenburg M. J. Van Ijzendoorn M. H. (2011a). Differential susceptibility to the environment: an evolutionary-neurodevelopmental theory. Dev. Psychopathol. 23, 7–28 10.1017/S0954579410000611 21262036

[B34] Ellis B. J. Shirtcliff E. A. Boyce W. T. Deardorff J. Essex M. J. (2011b). Quality of early family relationships and the timing and tempo of puberty: effects depend on biological sensitivity to context. Dev. Psychopathol. 23, 85–99 10.1017/S0954579410000660 21262041 PMC3033698

[B35] Ernst M. Daniele T. Frantz K. (2011). New perspectives on adolescent motivated behavior: attention and conditioning. Dev. Cogn. Neurosci. 1, 377–389 10.1016/j.dcn.2011.07.013 21977221 PMC3184422

[B36] Ernst M. Pine D. S. Hardin M. (2006). Triadic model of the neurobiology of motivated behavior in adolescence. Psychol. Med. 36, 299–312 10.1017/S0033291705005891 16472412 PMC2733162

[B37] Forbes E. E. Dahl R. E. (2010). Pubertal development and behavior: hormonal activation of social and motivational tendencies. Brain Cogn. 72, 66–72 10.1016/j.bandc.2009.10.007 19942334 PMC3955709

[B38] Forbes E. E. Phillips M. L. Ryan N. D. Dahl R. E. (2011). Neural systems of threat processing in adolescents: role of pubertal maturation and relation to measures of negative affect. Dev. Neuropsychol. 36, 429–452 10.1080/87565641.2010.550178 21516542 PMC3085008

[B39] Force U. (1996). Guide to Clinical Preventive Services, 2nd Edn (Alexandria, VA: International Medical Publishing).

[B40] Geier C. F. Terwilliger T. Velanova K. Luna B. (2010). Immaturities in reward processing and its influence on inhibitory control in adolescence. Cereb. Cortex 20, 1613–1629 10.1093/cercor/bhp225 19875675 PMC2882823

[B41] Giedd J. N. Blumenthal J. Jeffries N. O. Castellanos F. X. Liu H. Zijdenbos A. Paus T. Evans A. C. Rapoport J. L. (1999). Brain development during childhood and adolescence: a longitudinal MRI study. Nat. Neurosci. 2, 861–863 10.1038/13158 10491603

[B42] Giedd J. N. Clasen L. S. Lenroot R. Greenstein D. Wallace G. L. Ordaz S. Molloy E. A. Blumenthal J. D. Tossell J. W. Stayer C. Samango-Sprouse C. Shen D. Davatzikos C. Merke D. Chrousos G. P. (2006). Puberty-related influences on brain development. Mol. Cell. Endocrinol. 254-255, 154–162 10.1016/j.mce.2006.04.016 16765510

[B43] Grumbach M. M. Styne D. M. (2003). Puberty: ontogeny, neuroendocrinology, physiology, and disorders, in Williams Textbook of Endocrinology, 10th Edn eds Larsen P. R. Kronenberg H. M. Melmed S. Polonsky K. S. (New York, NY: Elsevier), 1115–1286

[B44] Hare T. A. Tottenham N. Galvan A. Voss H. U. Glover G. H. Casey B. J. (2008). Biological substrates of emotional reactivity and regulation in adolescence during an emotional Go-Nogo task. Biol. Psychiatry 63, 927–934 10.1016/j.biopsych.2008.03.015 18452757 PMC2664095

[B45] Hermans E. J. Ramsey N. F. Van Honk J. (2008). Exogenous testosterone enhances responsiveness to social threat in the neural circuitry of social aggression in humans. Biol. Psychiatry 63, 263–270 10.1016/j.biopsych.2007.05.013 17727825

[B46] Herting M. M. Maxwell E. C. Irvine C. Nagel B. J. (2011). The impact of sex, puberty, and hormones on white matter microstructure in adolescents. Cereb. Cortex 22, 1979–1992 10.1093/cercor/bhr246 22002939 PMC3412439

[B47] Jacobs E. D'esposito M. (2011). Estrogen shapes dopamine-dependent cognitive processes: implications for women's health. J. Neurosci. 31, 5286–5293 10.1523/JNEUROSCI.6394-10.2011 21471363 PMC3089976

[B48] Joinson C. Heron J. Araya R. Paus T. Croudace T. Rubin M. Marcus M. Lewis G. (2012). Association between pubertal development and depressive symptoms in girls from a UK cohort. Psychol. Med. 12, 1–11 10.1017/S003329171200061X 22717026

[B49] Ladouceur C. D. Dahl R. E. Williamson D. E. Birmaher B. Ryan N. D. Casey B. J. (2005). Altered emotional processing in pediatric anxiety disorder, depression, and co-morbid anxiety and depression. J. Abnorm. Child Psychol. 33, 165–177 10.1007/s10802-005-1825-z 15839495

[B50] Ladouceur C. D. Peper J. S. Dahl R. E. (2012). White matter development in adolescence: the influence of puberty and implications for affective disorders. Dev. Cogn. Neurosci. 2, 34–56 10.1016/j.dcn.2011.06.002 PMC3256931 22247751

[B51] Ladouceur C. D. Silk J. S. Dahl R. E. Ostapenko L. Kronhaus D. Phillips M. L. (2009). Fearful faces influence attentional control processes in anxious youth and adults. Emotion 9, 855–864 10.1037/a0017747 20001128

[B52] Larson R. W. Asmussen L. (1991). Anger, worry, and hurt in early adolescence: an enlarging world of negative emotions, in Adolescent stress: Causes and Consequences, eds Colten M. E. Gore S. (Hawthorne, NY: Aldine de Gruyter), 21–41

[B53] Lenroot R. K. Giedd J. N. (2010). Sex differences in the adolescent brain. Brain Cogn. 72, 46–55 10.1016/j.bandc.2009.10.008 19913969 PMC2818549

[B54] Lenroot R. K. Gogtay N. Greenstein D. K. Wells E. M. Wallace G. L. Clasen L. S. Blumenthal J. D. Lerch J. Zijdenbos A. P. Evans A. C. Thompson P. M. Giedd J. N. (2007). Sexual dimorphism of brain development trajectories during childhood and adolescence. Neuroimage 36, 1065–1073 10.1016/j.neuroimage.2007.03.053 17513132 PMC2040300

[B55] Lewis D. A. (1997). Development of the prefrontal cortex during adolescence: insights into vulnerable neural circuits in schizophrenia. Neuropsychopharmacology 16, 385–398 10.1016/S0893-133X(96)00277-1 9165494

[B56] Luna B. Garver K. Urban T. Lazar N. Sweeney J. (2004). Maturation of cognitive processes from late childhood to adulthood. Child Dev. 75, 1357–1372 10.1111/j.1467-8624.2004.00745.x 15369519

[B57] Manuck S. B. Marsland A. L. Flory J. D. Gorka A. Ferrell R. E. Hariri A. R. (2010). Salivary testosterone and a trinucleotide (CAG) length polymorphism in the androgen receptor gene predict amygdala reactivity in men. Psychoneuroendocrinology 35, 94–104 10.1016/j.psyneuen.2009.04.013 19493626 PMC2825741

[B58] Marshall W. A. Tanner J. M. (1969). Variations in pattern of pubertal changes in girls. Arch. Dis. Child 44, 291–303 5785179 10.1136/adc.44.235.291 PMC2020314

[B59] Marshall W. A. Tanner J. M. (1970). Variations in the pattern of pubertal changes in boys. Arch. Dis. Child 45, 13–23 5440182 10.1136/adc.45.239.13 PMC2020414

[B60] Martin C. A. Kelly T. H. Rayens M. K. Brogli B. R. Brenzel A. Smith W. J. Hatim A. O. (2002). Sensation seeking, puberty, and nicotine, alcohol, and marijuana use in adolescence. J. Am. Acad. Child Adolesc. Psychiatry 41, 1495–1502 10.1097/00004583-200212000-00022 12447037

[B61] Mayberg H. (2001). Depression and frontal-subcortical circuits: focus on prefrontal-limbic interactions, in Frontal-Subcortical Circuits in Psychiatric and Neurological Disorders, eds Lichter D. G. Cummings J. L. (New York, NY: The Guilford Press), 177–206

[B62] Mcclure E. B. Monk C. S. Nelson E. E. Parrish J. M. Adler A. D. Blair R. J. Fromm S. Charney D. S. Leibenluft E. Ernst M. Pine D. S. (2007). Abnormal attention modulation of fear circuit function in pediatric generalized anxiety disorder. Arch. Gen. Psychiatry 64, 97–106 10.1001/archpsyc.64.1.97 17199059

[B63] Mehta P. H. Beer J. (2010). Neural mechanisms of the testosterone-aggression relation: the role of orbito-frontal cortex. J. Cogn. Neurosci. 22, 2357–2368 10.1162/jocn.2009.21389 19925198

[B64] Mendle J. Harden K. P. Brooks-Gunn J. Graber J. A. (2010). Development's tortoise and hare: pubertal timing, pubertal tempo, and depressive symptoms in boys and girls. Dev. Psychol. 46, 1341–1353 10.1037/a0020205 20822243 PMC3114603

[B65] Mendle J. Harden K. P. Brooks-Gunn J. Graber J. A. (2012). Peer relationship and depressive symptomatology in boys at puberty. Dev. Psychol. 48, 429–435 10.1037/a0026425 22103302 PMC3714849

[B66] Merikangas K. R. Akiskal H. S. Angst J. Greenberg P. E. Hirschfeld R. M. Petukhova M. Kessler R. C. (2007). Lifetime and 12-month prevalence of bipolar spectrum disorder in the National Comorbidity Survey replication. Arch. Gen. Psychiatry 64, 543–552 10.1001/archpsyc.64.5.543 17485606 PMC1931566

[B67] Monk C. S. Klein R. G. Telzer E. H. Schroth E. A. Mannuzza S. Moulton Iii J. L. Guardino M. Mcclure-Tone E. B. Fromm S. Blair J. R. Pine D. S. Ernst M. (2008). Amygdala and nucleus accumbens activation to emotional facial expressions in children and adolescents at risk for major depression. Am. J. Psychiatry 165, 90–98 10.1176/appi.ajp.2007.06111917 17986682

[B68] Monk C. S. Mcclure E. B. Nelson E. E. Zarahn E. Bilder R. M. Leibenluft E. Charney D. S. Ernst M. Pine D. S. (2003). Adolescent immaturity in attention-related brain engagement to emotional facial expressions. Neuroimage 20, 420–428 10.1016/S1053-8119(03)00355-0 14527602

[B69] Moore III W. E. Pfeifer J. H. Masten C. L. Mazziotta J. C. Iacoboni M. Dapretto M. (2012). Facing puberty: associations between pubertal development and neural responses to affective facial displays. Soc. Cogn. Affect. Neurosci. 7, 35–43 10.1093/scan/nsr066 22228752 PMC3252633

[B70] Mueller S. C. (2011). The influence of emotion on cognitive control: relevance for development and adolescent psychopathology. Front. Psychol. 2:327 10.3389/fpsyg.2011.00327 22275904 PMC3223617

[B71] Naftolin F. Malaspina D. (2007). Estrogen, estrogen treatment and the post-reproductive woman's brain. Maturitas 20, 23–26 10.1016/j.maturitas.2007.02.005 17391878

[B72] Natsuaki M. N. Klimes-Dougan B. Ge X. Shirtcliff E. A. Hastings P. D. Zahn-Waxler C. (2009). Early pubertal maturation and internalizing problems in adolescence: sex differences in the role of cortisol reactivity to interpersonal stress. J. Clin. Child Adolesc. Psychol. 38, 513–524 10.1080/15374410902976320 20183638 PMC3061854

[B73] Nelson E. E. Leibenluft E. Mcclure E. Pine D. S. (2005). The social-orientation of adolescence: a neuroscience perspective on the process and its relation to psychopathology. Psychol. Med. 35, 163–174 15841674 10.1017/s0033291704003915

[B74] Neufang S. Specht K. Hausmann M. Gunturkun O. Herpertz-Dahlmann B. Fink G. R. Konrad K. (2009). Sex differences and the impact of steroid hormones on the developing human brain. Cereb. Cortex 19, 464–473 10.1093/cercor/bhn100 18550597

[B75] Op De Macks Z. A. Moor B. G. Overgaauw S. Guroglu B. Dahl R. E. Crone E. A. (2011). Testosterone levels correspond with increased ventral striatum activation in response to monetary rewards in adolescents. Dev. Cogn. Neurosci. 1, 506–516 10.1016/j.dcn.2011.06.003 22436568 PMC6987540

[B76] Ozer E. M. Macdonald T. Irwin C. E. Jr. (2002). Adolescent health care in the United States: implications and projections for the new millennium, in The Changing Adolescent Experience: Societal Trends and the Transition to Adulthood, eds Mortimer J. T. Larson R. W. (New York, NY: Cambridge University Press), 129–174

[B77] Parker L. N. (1991). Adrenarche. Endocrinol. Metab. Clin. Am. 20, 71–83 2029889

[B78] Paus T. (2010). Growth of white matter in the adolescent brain: myelin or axon? Brain Cogn. 72, 26–35 10.1016/j.bandc.2009.06.002 19595493

[B79] Pavuluri M. N. O'connor M. M. Harral E. M. Sweeney J. A. (2008). An fMRI study of the interface between affective and cognitive neural circuitry in pediatric bipolar disorder. Psychiatry Res. 162, 244–255 10.1016/j.pscychresns.2007.10.003 18294820 PMC2323905

[B80] Peper J. S. Brouwer R. M. Schnack H. G. Van Baal G. C. Van Leeuwen M. Van Den Berg S. M. Delemarre-Van De Waal H. A. Boomsma D. I. Kahn R. S. Hulshoff Pol H. E. (2009). Sex steroids and brain structure in pubertal boys and girls. Psychoneuroendocrinology 34, 332–342 10.1016/j.psyneuen.2008.09.012 18980810

[B81] Peper J. S. Brouwer R. M. Schnack H. G. Van Baal G. C. Van Leeuwen M. Van Den Berg S. M. Delemarre-Van De Waal H. A. Janke A. L. Collins D. L. Evans A. C. Boomsma D. I. Kahn R. S. Hulshoff Pol H. E. (2008). Cerebral white matter in early puberty is associated with luteinizing hormone concentrations. Psychoneuroendocrinology 33, 909–915 10.1016/j.psyneuen.2008.03.017 18640784

[B82] Peper J. S. Pol H. E. H. Crone E. A. Van Honk J. (2011). Sex steroids and brain structure in pubertal boys and girls: a mini-review of neuroimaging studies. Neuroscience 191, 28–37 10.1016/j.neuroscience.2011.02.014 21335066

[B83] Perrin J. S. Herve P. Y. Leonard G. Perron M. Pike G. B. Pitiot A. Richer L. Veillette S. Pausova Z. Paus T. (2008). Growth of white matter in the adolescent brain: role of testosterone and androgen receptor. J. Neurosci. 28, 9519–9524 10.1523/JNEUROSCI.1212-08.2008 18799683 PMC6671132

[B83a] Pfaff D. W. Keiner M. (1973). Atlas of estradiol-concentrating neurons in the central nervous system of the female rat. J. Comp. Neurol. 151, 121–158 10.1002/cne.901510204 4744471

[B84] Phillips M. L. Drevets W. C. Rauch S. L. Lane R. (2003). Neurobiology of emotion perception II: implications for major psychiatric disorders. Biol. Psychiatry 54, 515–528 10.1016/S0006-3223(03)00171-9 12946880

[B85] Phillips M. L. Ladouceur C. D. Drevets W. C. (2008). A neural model of voluntary and automatic emotion regulation: implications for understanding the pathophysiology and neurodevelopment of bipolar disorder. Mol. Psychiatry 13, 833–857 10.1038/mp.2008.65 18574483 PMC2745893

[B86] Pine D. S. Cohen P. Gurley D. Brook J. Ma Y. (1998). The risk for early-adulthood anxiety and depressive disorders in adolescents with anxiety and depressive disorders. Arch. Gen. Psychiatry 55, 56–64 9435761 10.1001/archpsyc.55.1.56

[B87] Price J. L. Drevets W. C. (2010). Neurocircuitry of mood disorders. Neuropsychopharmacology 35, 192–216 10.1038/npp.2009.104 19693001 PMC3055427

[B88] Raznahan A. Lee Y. Stidd R. Long R. Greenstein D. Clasen L. Addington A. Gogtay N. Rapoport J. L. Giedd J. N. (2010). Longitudinally mapping the influence of sex and androgen signaling on the dynamics of human cortical maturation in adolescence. Proc. Natl. Acad. Sci. U.S.A. 107, 16988–16993 10.1073/pnas.1006025107 20841422 PMC2947865

[B89] Reyna V. F. Farley F. (2006). Risk and rationality in adolescent decision making: implications for theory, practice, and public policy. Psychol. Sci. Pub. Interest 7, 1–44 10.1111/j.1529-1006.2006.00026.x 26158695

[B90] Sarkey S. Azcoitia I. Garcia-Segura L. M. Garcia-Ovejero D. Doncarlos L. L. (2008). Classical androgen receptors in non-classical sites in the brain. Horm. Behav. 53, 753–764 10.1016/j.yhbeh.2008.02.015 18402960 PMC2413135

[B91] Savitz J. Drevets W. C. (2009). Bipolar and major depressive disorder: neuroimaging the developmental-degenerative divide. Neurosci. Biobehav. Rev. 33, 699–771 10.1016/j.neubiorev.2009.01.004 19428491 PMC2858318

[B92] Schulz K. M. Molenda-Figueira H. Sisk C. (2009). Back to the future: the organizational-activational hypothesis adapted to puberty and adolescence. Horm. Behav. 55, 597–604 10.1016/j.yhbeh.2009.03.010 19446076 PMC2720102

[B93] Shirtcliff E. A. Dahl R. E. Pollak S. D. (2009). Pubertal development: correspondence between hormonal and physical development. Child Dev. 80, 327–337 10.1111/j.1467-8624.2009.01263.x 19466995 PMC2727719

[B94] Siegle G. J. Steinhauer S. R. Thase M. E. (2004). Pupillary assessment and computational modeling of the Stroop task in depression. Int. J. Psychophysiol. 52, 63–76 10.1016/j.ijpsycho.2003.12.010 15003373

[B95] Silk J. S. Davis S. Mcmakin D. L. Dahl R. E. Forbes E. E. (2012a). Why do anxious children become depressed teenagers? The role of social evaluative threat and reward processing. Psychol. Med. 17, 1–13 10.1017/S0033291712000207 22340187 PMC3360132

[B96] Silk J. S. Stroud L. R. Siegle G. J. Dahl R. E. Lee K. H. Nelson E. E. (2012b). Peer acceptance and rejection through the eyes of youth: pupillary, eyetracking and ecological data from the Chatroom Interact task. Soc. Cogn. Affect. Neurosci. 7, 93–105 10.1093/scan/nsr044 21775386 PMC3252631

[B97] Silk J. S. Siegle G. J. Whalen D. J. Ostapenko L. J. Ladouceur C. D. Dahl R. E. (2009). Pubertal changes in emotional information processing: pupillary, behavioral, and subjective evidence during emotional work identification. Dev. Psychopathol. 21, 7–26 10.1017/S0954579409000029 19144220 PMC2629078

[B98] Simerly R. B. Chang C. Muramatsu M. Swanson L. W. (1990). Distribution of androgen and estrogen receptor mRNA-containing cells in the rat brain: an *in situ* hybridation study. J. Comp. Neurol. 294, 76–95 10.1002/cne.902940107 2324335

[B99] Sisk C. L. Zehr J. L. (2005). Pubertal hormones organize the adolescent brain and behavior. Front. Neuroendocrinol. 26, 163–174 10.1016/j.yfrne.2005.10.003 16309736

[B100] Smith A. B. Halari R. Giampetro V. Brammer M. Rubia K. (2011). Developmental effects of reward on sustained attention networks. Neuroimage 56, 1693–1704 10.1016/j.neuroimage.2011.01.072 21300162

[B101] Sowell E. R. Petersen B. S. Thompson P. M. Welcome S. E. Henkenius A. L. Toga A. W. (2003). Mapping cortical change across the human life span. Nat. Neurosci. 6, 309–315 10.1038/nn1008 12548289

[B102] Spear L. P. (2000). The adolescent brain and age-related behavioral manifestations. Neurosci. Biobehav. Rev. 24, 417–463 10.1016/S0149-7634(00)00014-2 10817843

[B103] Spear L. P. (2010). The Behavioral Neuroscience of Adolescence, (New York, NY: WW Norton and Company Inc.)

[B104] Stanton S. J. Wirth M. M. Waugh C. E. Schultheiss O. C. (2009). Endogenous testosterone levels are associated with amygdala and ventromedial prefrontal cortex responses to anger faces in men but not women. Biol. Psychol. 81, 118–122 10.1016/j.biopsycho.2009.03.004 19428976 PMC2691609

[B105] Steinberg L. (2005). Cognitive and affective development in adolescence. Trends Cogn. Sci. 9, 69–74 10.1016/j.tics.2004.12.005 15668099

[B106] Steinberg L. (2007). Risk-taking in adolescence: new perspectives from brain and behavioral science. Curr. Direct. Psychol. Sci. 16, 55–59

[B107] Styne D. Grumbach M. (2002). Puberty in boys and girls, in Hormones, Brain, and Behavior, eds Pfaff D. Arnold A. Etgen A. Fahrbach S. Rubin. R. (San Francisco, CA: Academic Press), 661–716

[B108] Tottenham N. Hare T. A. Casey B. J. (2011). Behavioral assessment of emotion discrimination, emotion regulation, and cognitive control in childhood, adolescence, and adulthood. Front. Psychol. 2:39 10.3389/fpsyg.2011.00039 21716604 PMC3110936

[B109] Van Wingen G. A. Ossewaarde L. Backstrom T. Hermans E. J. Fernandez G. (2011). Gonadal hormone regulation of the emotion circuitry in humans. Neuroscience 191, 38–45 10.1016/j.neuroscience.2011.04.042 21540080

[B110] Van Wingen G. A. Zylicz S. A. Pieters S. Mattern C. Verkes R. J. Buitelaar J. K. Fernandez G. (2010). Testosterone reduces amygdala-orbitofrontal cortex coupling. Psychoneuroendocrinology 35, 105–113 10.1016/j.psyneuen.2009.09.007 19782476

[B111] Vermeersch H. T'sjoen T. Kaufman J. Vincke J. (2008). The role of testosterone in aggressive and non-aggressive risk-taking behavior in adolescent boys. Horm. Behav. 53, 463–471 10.1016/j.yhbeh.2007.11.021 18234200

[B112] Volman I. Toni I. Verhagen L. Roelofs K. (2011). Endogenous testosterone modulates prefrontal-amygdala connectivity during social emotional behavior. Cereb. Cortex 21, 2282–2290 10.1093/cercor/bhr001 21339377 PMC3169658

[B113] Wahlstrom D. White T. Luciana M. (2010). Neurobehavioral evidence for changes in dopamine system activity during adolescence. Neurosci. Biobehav. Rev. 34, 631–648 10.1016/j.neubiorev.2009.12.007 20026110 PMC2845533

[B114] Walf A. A. Frye C. A. (2006). A review and update of mechanisms of estrogen in the hippocampus and amygdala for anxiety and depression behavior. Neuropsychopharmacology 31, 1097–1111 10.1038/sj.npp.1301067 16554740 PMC3624621

[B115] Worthman C. (1999). Epidemiology of human development, in Hormones, Health, and Behavior: A Socio-Ecological and Lifespan Perspectives, eds Panter-Brick C. Worthman C. (New York, NY: Cambridge University Press), 47–104

